# Increasing the performance of a superconducting spin valve using a Heusler alloy

**DOI:** 10.3762/bjnano.9.167

**Published:** 2018-06-12

**Authors:** Andrey A Kamashev, Aidar A Validov, Joachim Schumann, Vladislav Kataev, Bernd Büchner, Yakov V Fominov, Ilgiz A Garifullin

**Affiliations:** 1 Zavoisky Physical-Technical Institute, Russian Academy of Sciences, 420029 Kazan, Russia; 2 Leibniz Institute for Solid State and Materials Research IFW Dresden, D-01171 Dresden, Germany; 3 Institute for Solid State Physics, Technical University Dresden, D-01062 Dresden, Germany; 4 L. D. Landau Institute for Theoretical Physics, Russian Academy of Sciences, 142432 Chernogolovka, Russia; 5 National Research University Higher School of Economics, 101000 Moscow, Russia

**Keywords:** ferromagnet, proximity effect, spin valve, superconductor

## Abstract

We have studied superconducting properties of spin-valve thin-layer heterostructures CoO*_x_*/F1/Cu/F2/Cu/Pb in which the ferromagnetic F1 layer was made of Permalloy while for the F2 layer we have taken a specially prepared film of the Heusler alloy Co_2_Cr_1−_*_x_*Fe*_x_*Al with a small degree of spin polarization of the conduction band. The heterostructures demonstrate a significant superconducting spin-valve effect, i.e., a complete switching on and off of the superconducting current flowing through the system by manipulating the mutual orientations of the magnetization of the F1 and F2 layers. The magnitude of the effect is doubled in comparison with the previously studied analogous multilayers with the F2 layer made of the strong ferromagnet Fe. Theoretical analysis shows that a drastic enhancement of the switching effect is due to a smaller exchange field in the heterostructure coming from the Heusler film as compared to Fe. This enables to approach an almost ideal theoretical magnitude of the switching in the Heusler-based multilayer with a F2 layer thickness of ca. 1 nm.

## Introduction

Historically, the first concept to manipulate the transition temperature *T*_c_ of a superconductor by sandwiching it between two ferromagnetic insulators was thought of by de Gennes [1]. Regarding the case of metallic ferromagnets, the physical principle of a superconducting spin valve (SSV) is based on the idea proposed by Oh et al. in 1997 [2] who calculated the pairing wave-function amplitude in a trilayer F1/F2/S (where F1 and F2 are ferromagnetic layers and S is a superconducting layer) and found out that the superconducting (SC) transition temperature *T*_c_ depends on the mutual orientation of the magnetizations **M**_1_ and **M**_2_ of the layers F1 and F2. Later, another construction based on three-layer thin films F1/S/F2 was proposed also theoretically [3–4]. According to the above theories, for the parallel (P) configuration of **M**_1_ and **M**_2_ the transition temperature 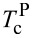 should be always smaller than 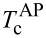 for the antiparallel (AP) orientation of the magnetic vectors. This is because in the former case the mean exchange field from the F-layers destructively acting on the Cooper pairs is larger. Thus, under favorable conditions the switching between AP and P configurations, which could be achieved by an appropriate application of a small external magnetic field, should yield a complete switching on and off of the superconducting current in such a construction.

A number of experimental studies have confirmed the predicted effect of the mutual orientation of magnetizations in the F1/S/F2 structure on *T*_c_ [5–9]. However, the major difficulty in a practical realization of an SSV, i.e., to obtain a difference between 
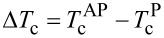
 larger than the width δ*T*_c_ of the superconducting transition for a given configuration of **M**_1_ and **M**_2_, was not overcome in these works. One should note that the reported antiferromagnetically coupled [Fe/V]*_n_* superlattice [10] in which Δ*T*_c_ could implicitly reach up to 200 mK cannot be considered as an SSV because this system can not be switched from the AP to P orientation of the magnetizations instantaneously.

In addition to that, the SSV effect becomes more complicated due to the following fact [11]: It is well known [12] that in the ferromagnetic layer the Cooper pair acquires a nonzero momentum due to the Zeeman splitting of electronic levels. Its wave function oscillates in space when moving away from the S/F interface. If the F layer is thin enough, the wave function is reflected from the surface opposite to the S/F interface. The interference of the incident and reflected functions arises. Depending on the thickness of the F layer, the interference at the S/F interface can be constructive or destructive. This should lead to an increase or decrease of the *T*_c_ of the S/F structure depending on the interference type.

From the experimental point of view, the results obtained for both theoretical designs of the SSV suggested that the scheme by Oh et al. [2] may be the most promising for the realization of the full SSV effect. Indeed, this approach turns out to be successful. Previously we have demonstrated a full switching between the normal and uperconducting states for the CoO*_x_*/Fe1/Cu/Fe2/In spin-valve structure [13]. Later on we replaced the superconducting In by Pb in order to improve superconducting parameters [14] and introduced an additional technical Cu interlayer (N2) in order to prevent degradation of the samples [15]. Thus, the final design of the SSV structures was set as AFM/F1/N1/F2/N2/S. In this construction the Cu interlayer (N1) decouples magnetizations of the Fe1 (F1) and Fe2 (F2) layers and the antiferromagnetic (AFM) CoO*_x_* layer biases the magnetization of the Fe1 layer by anisotropy fields. Despite substantial experimental efforts in optimizing the properties of the In- and Pb-based SSVs [16–17], in particular in reducing the width δ*T*_c_, our theoretical analysis of the properties of such multilayers in the framework of the theory of [11] has shown that the experimentally achieved magnitude of Δ*T*_c_ of the SSV effect of 20 mK and 40 mK for the two types of the S layer, respectively, was substantially smaller as expected on theoretical grounds. Recently the interest on SSVs increased considerably (see the review in [18] and the very recent publications [19–24]).

Here, we present experimental results that evidence a significant improvement of the magnitude of Δ*T*_c_ in a Pb-based SSV by using the ferromagnetic Heusler alloy (HA) Co_2_Cr_1−_*_x_*Fe*_x_*Al as a material for the F2 layer. Prepared under well-defined conditions [25] the HA layer produces a substantially smaller exchange field acting on the superconducting Cooper pairs as compared to the Fe layer of the same thickness. This opens a possibility to grow heterostructures where the theoretically desired parameters for the maximum SSV effect could be practically realized yielding the doubling of the magnitude of the SSV effect up to the almost ideal theoretical value.

## Results

**Figure 1:**
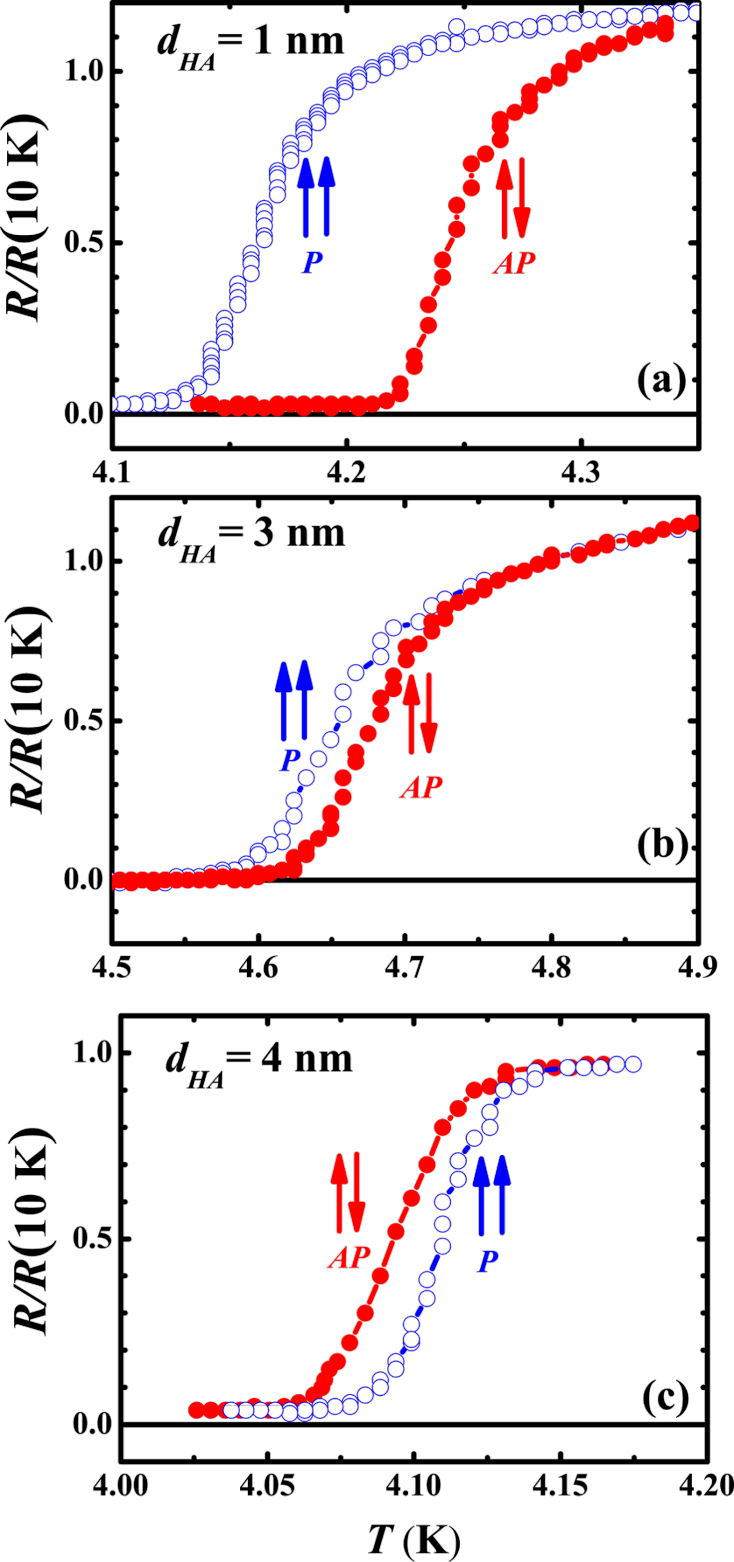
Superconducting transition curves for CoO*_x_*/Py(5)/Cu(4)/Co_2_Cr_1−_*_x_*Fe*_x_*Al/Cu(1.5)/Pb(50) multilayers with different thicknesses of the HA layer *d*_HA_ for P (open circles) and AP (closed circles) mutual orientation of the magnetizations **M**_1_ and **M**_2_ of the Py and Co_2_Cr_1−_*_x_*Fe*_x_*Al*_x_* ferromagnetic layers, respectively: (a) *d*_HA_ = 1 nm; (b) *d*_HA_ = 3 nm; (c) *d*_HA_ = 4 nm.

Technical particularities of the fabrication of the SSV heterostructures that have been studied in the present work have been reported in detail previously (see Supporting Information File 1). The new HA-based part of the multilayer F2/N2/S = HA/Cu/Pb has been investigated in detail with the focus on the S/F proximity effect very recently. It was shown [25] that the degree of the spin polarization of the conduction band of the HA film amounts to 30% for the films prepared at a particular substrate temperature of *T*_sub_ = 300 K during the growth of the HA layer and to 70% at *T*_sub_ = 600 K. In the AFM/F1/N1/F2/N2/S structure it would be advantageous to achieve a penetration depth of the Cooper pairs into F2 ferromagnetic layer as large as possible. This means that the spin polarization of the conduction band should be small. To fulfill this requirement we have prepared a set of samples CoO*_x_*/Py(5 nm)/Cu(4 nm)/Co_2_Cr_1−_*_x_*Fe*_x_*Al/Cu(1.5 nm)/Pb(50 nm) with the HA layer of different thickness grown at *T*_sub_ = 300 K. Representative superconducting transition curves are shown in Figure 1.

**Figure 2:**
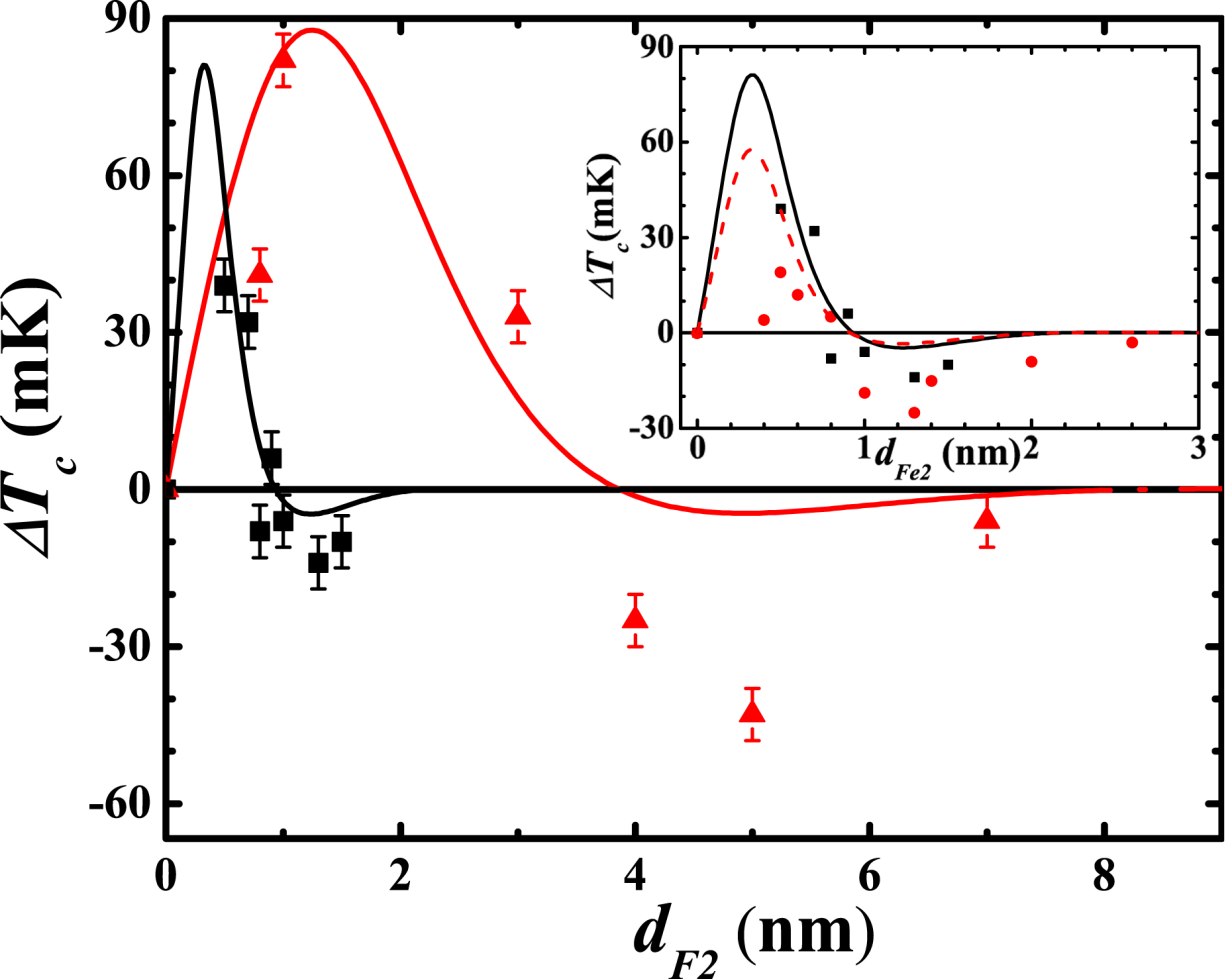
Dependence of 
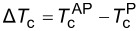
 on the thickness of layer F2, *d*_F2_ in the SSV heterostructures AFM/F1/N1/F2/N2/S. Triangles are the data points for CoO*_x_*/Py(5)/Cu(4)/Co_2_Cr_1−_*_x_*Fe*_x_*Al*_x_*/Cu(1.5)Pb(50) from the present work. For comparison previous results for CoO*_x_*/Fe1/Cu/Fe2/Cu/Pb multilayers [17] are plotted with squares in the main panel and in the insert in which, additionally, the data for the CoO*_x_*/Fe1/Cu/Fe2/In SSV from [16] are plotted with circles for comparison. Solid and dashed lines present the results of theoretical modeling.

A clear shift of the curves upon switching the mutual orientation of the magnetizations **M**_1_ and **M**_2_ of the ferromagnetic layers between P and AP configurations characteristic of the SSV effect is clearly visible. The superconducting transition temperature was determined as a midpoint of the transition curve. The dependence of the magnitude Δ*T*_c_ of the SSV effect on the thickness of the HA layer is presented in Figure 2.

The dependence Δ*T*_c_(*d*_HA_) reveals an oscillating behavior due to the interference of the Cooper pair wave functions reflected from both surfaces of the ferromagnetic F2 layer (of the order of 4 nm) proximate to the superconducting layer. This yields for certain thicknesses of the F2 layer an inverse SSV effect Δ*T*_c_ *<* 0 [26]. The most remarkable result of the present study is the magnitude of the direct SSV effect, which reaches for *d*_HA_ = 1 nm (about two monolayers of HA) the maximum value of 80 mK (triangles in Figure 2). This surpasses the result for the analogous heterostructure with Fe as the F2 layer [17] by a factor of 2 (see the data comparison in Figure 2). As we will discuss below, the achieved SSV effect in the Pb-based heterostructure with the HA layer approaches the maximum value predicted by theory. The scattering of Δ*T*_c_ is mainly due to some uncertainty in the determination of the thickness of the HA layer, which indirectly affects the accuracy of the determination of Δ*T*_c_.

## Discussion

To set up the basis for discussion we fist summarize the parameters of the theory [11] describing the SSV effect in the above systems. As described in [17], in order to estimate these parameters characterizing the properties of the S layer we use our experimental data on the resistivity and on the dependence of *T*_c_ on the S-layer thickness at a large unchanged thickness of the F layer in the S/F bilayer. The residual resistivity ρ_S_ = ρ(*T*_c_) can be determined from the residual resistivity ratio RRR = *R*(*T* = 300 K)/*R*(*T*_c_= [ρ(300 K) + ρ(*T*_c_)]/ρ(*T*_c_). Since the room-temperature resistivity of the Pb layer is dominated by the phonon contribution ρ_ph_(300 K) = 21 μΩ·cm [27] we obtain the ρ_S_ values presented below in Table 1. Then with the aid of the Pippard relations [28] the following equality can be obtained [17]:

[1]
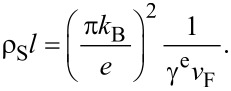


Here γ^e^ denotes the electronic specific heat coefficient, *v*_F_ is the Fermi velocity of the conduction electrons, and *l* is the mean-free path of the conduction electrons. Using for Pb γ^e^ = 1.6 × 10^3^ erg/K^2^·cm^3^ [27], from Equation 1 one can find the mean-free path *l*_S_, the diffusion coefficient of conduction electrons *D*_S_ and the superconducting coherence length

[2]
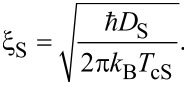


The same procedure can be applied for the F layers taking into account the definition of the superconducting coherence length in the F layers [29],

[3]
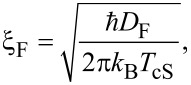


where *D*_F_ is the diffusion coefficient for the conduction electrons in the F layer and *T*_cS_ is the superconducting transition temperature for an isolated S layer.

The theory contains also the material-specific parameter γ and the interface transparency parameter γ*_b_*. The first one is defined as

[4]
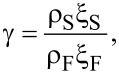


the second one can be calculated from the critical thickness of the S layer, 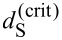, which is defined as the thickness below which there is no superconductivity in the S/F bilayer: 
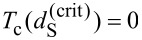
.

In the limiting case 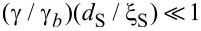, the thickness 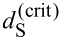 can be calculated explicitly as [29]

[5]
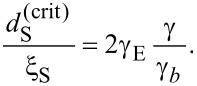


Here γ_E_ ≈ 1.78 is the Euler constant. Our data yield 
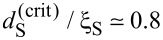
 (γ*_b_* = 1.95) for the Fe/In system, 
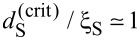
 (γ*_b_* = 2.7) for the Fe/Cu/Pb system, and 
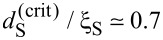
 (γ*_b_* = 0.37) for the HA/Cu/Pb system. All obtained parameters are presented in Table 1. The larger value of γ*_b_* for the Fe/Cu/Pb system compared to the Ha/Cu/Pb system makes, of course, sense. Indeed, the difference between the HA system and the Fe system is seen in a difference of γ*_b_*, which helps to rationalize the use of a weaker F-layer.

**Table 1:** Parameters used for fitting of the theory to the experimental results [17].

parameter	1	2	3
	Fe2/In	Fe2/Cu/Pb	HA/Cu/Pb

ρ_S_, μΩ·cm	0.2	1.47	1.47
*l*_S_, nm	300	17	17
*D*_S_, cm^2^/s	1100	100	100
ξ_S_, nm	170	41	41
ρ_F_, μΩ·cm	10	10	130
*l*_F_, nm	10	10	6.41
*D*_F_, cm^2^/s	3.3	3.3	21.4
ξ_F_, nm	7.5	7.5	14
ξ*_h_*, nm	0.5	0.3	1.25
γ	0.45	0.78	0.03
γ*_b_*	1.95	2.7	0.37

Figure 2 summarizes the experimental values of Δ*T*_c_(*d*_F2_) for SSV heterostructures with HA as the F2 layer obtained in the present work and our previous results on Fe-based SSVs [16–17]. Solid lines in Figure 2 are theoretical results using the parameters listed in Table 1. The general feature of the SSVs with F2 = Fe is that the measured points at small thicknesses *d*_F2_ lie much lower than the theoretically expected positive maximum of Δ*T*_c_ (direct SSV effect). One should note that the difference in the theoretical maximum values of Δ*T*_c_ for In- and Pb-based systems (inset in Figure 2) is caused by the different values of the superconducting transition temperature of the single S layer (*T*_c_ = 3.4 K for In and *T*_c_ = 7.18 K for Pb). Obviously in both cases, to reach the expected maximum it would be necessary to further decrease the thickness of the F2 layer. It should be emphasized that the thickness *d*_F2_ is one of the crucial parameters for the functionality of the spin valve. It determines the number of the Cooper pairs that experience the influence of the exchange fields of both F layers in the heterostructure. In general, to get the maximum magnitude of the spin-valve effect Δ*T*_c_, the thickness *d*_F2_ of the F2 layer proximate to the S layer should be of the order or smaller than the penetration depth of the Cooper pairs into the F2 layer, 
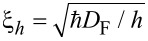
. Here *h* is the exchange splitting of the conduction band of a ferromagnet. The thinner the F2 layer is, the more Cooper pairs can reach the contact region between the two ferromagnetic layers where at certain thicknesses of the F1- and F2-layers the compensation effect of the exchange fields can take place in the AP case. For the previously studied Fe-based systems, *h* was of the order of 1 eV and ξ*_h_* amounted to 0.6–0.8 nm [16–17]. According to theory [11], this means that the maximum of Δ*T*_c_ should occur in the interval between 0.3 and 0.4 nm (inset of Figure 2). With the available experimental setup, it is practically impossible to grow a continuous iron film of such small thickness.

It is well known [30–31] that in dilute alloys, e.g., in PdNi alloys with 10% of Ni, ξ*_h_* is of the order of 5 nm, which is an order of magnitude larger compared to pure ferromagnetic elements such as Fe, Ni, or Co. As our present experimental results demonstrate, the use of a Heusler alloy for the growth of the F2 layer is very beneficial. It greatly relaxes the stringent condition on the minimum thickness of the F2 layer. Indeed, according to the previous analysis of the Pb/Cu/Co_2_Cr_1−_*_x_*Fe*_x_*Al trilayers, the HA film grown at the substrate temperature of *T*_sub_ = 300 K can be classified as a weak ferromagnet with a relatively small exchange field *h*^HA^ ≈ 0.2 eV [25]. As can be seen in Figure 2, this reduction of *h* shifts the peak of the theoretical values of Δ*T*_c_(*d*_F2_) for F2 = HA to larger thicknesses of the order of 1 nm, which can be easily reached experimentally. Under these conditions the measured maximum magnitude of Δ*T*_c_ is two times larger compared to the best previous result on the Fe-based SSVs (Figure 2). In fact, it almost reaches the theoretically predicted value suggesting that further optimization of the properties of the F2 layer is unlikely to significantly increase the SSV effect. In this respect it would be very interesting to explore theoretically and experimentally the option of optimization of the F1 layer in the SSV AFM/F1/N1/F2/N2/S heterostructure.

Recently, Singh et al. [32] reported a huge SSV effect for a S/F1/N/F2 structure made of amorphous MoGe, Ni, Cu and CrO_2_ as S, F1, N and F2, respectively. This structure exhibited a Δ*T*_c_ of ca. 1 K when changing the relative orientation of magnetizations of two F layers. The reason for such a surprisingly strong SSV effect remains unclear [33]. Gu et al. [34–35] reported Δ*T*_c_ ≈ 400 mK for three-layered Ho/Nb/Ho films.

Finally, a discrepancy between the theoretical curves and experimental data at larger thicknesses *d*_F2_ in the regime of the inverse (negative) SSV effect found for all the above discussed systems (Figure 2) needs to be commented. In this respect, we note that the assumptions of theory [11] do not fully comply with the properties of our samples. While the assumption of F layers being weak ferromagnets (exchange energy much smaller than the Fermi energy) is satisfied for the Heusler alloy, iron is closer to the limit of strong ferromagnets (exchange energy starts to be comparable with the Fermi energy). Accurate theoretical description of ferromagnets with large exchange splitting requires taking into account different densities of states in different spin subbands and modified boundary conditions at SF interfaces [36–37]. At the same time, the major inconsistency between theory and experiment in our case is probably related to the assumption of the dirty limit (mean free path much smaller than the coherence length). In our samples, these assumptions are close to the border of applicability or even not satisfied (depending on the specific material). The ferromagnets turn out to be strong enough so that the condition 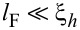 is not satisfied at all. Therefore, we cannot expect theory [11] to describe quantitative details of our results. Still, we observe that the theory captures main qualitative features of the experiment.

## Conclusion

In summary, we have experimentally demonstrated that using for the F2 layer in a CoO*_x_*/F1/Cu/F2/Cu/Pb heterostructure a specially prepared thin film of the Heusler alloy Co_2_Cr_1−_*_x_*Fe*_x_*Al with a small degree of the spin polarization of the conduction band significantly increases the magnitude of the superconducting spin valve effect Δ*T*_c_ as compared to similar systems with the F2 layer made of the strong ferromagnet Fe. It follows from our theoretical analysis that the experimentally achieved value is close to the maximum predicted by theory. The use of the Heusler alloy did not increase this maximum value beyond the theoretical result but enables to reach experimentally the maximum possible value of Δ*T*_c_ at a larger, technically realizable thickness of the F2 layer, in a full agreement with theory. It seems unlikely that further optimization of the material of the F2 layer would yield substantially larger values of Δ*T*_c_. An interesting alternative would be to optimize the parameters of the F1 layer, which is tempting to explore in the future.

## Supporting Information

File 1 Fabrication of the SSV heterostructures.
