# Supplementary material for: Increasing the performance of a superconducting spin valve using a Heusler alloy
Source: Beilstein J Nanotechnol. 2018 Jun 12;9:1764–9. doi: 10.3762/bjnano.9.167 (PMC6009251; doi:10.3762/bjnano.9.167)
Supplement: File 1 — Fabrication of the SSV heterostructures. [file Beilstein_J_Nanotechnol-09-1764-s001.pdf]

Supporting Information

for

Increasing the performance of a superconducting spin valve using a Heusler alloy

Andrey A. Kamashev[1,2], Aidar A. Validov[1], Joachim Schumann[2], Vladislav Kataev[2], Bernd Büchner[2,3], Yakov V. Fominov[4,5] and Ilgiz A. Garifullin*[1]

Address: [1]Zavoisky Physical-Technical Institute, Russian Academy of Sciences, 420029 Kazan, Russia; [2]Leibniz Institute for Solid State and Materials Research IFW Dresden, D-01171 Dresden, Germany; [3]Institute for Solid State Physics, Technical University Dresden, D-01062 Dresden, Germany; [4]L. D. Landau Institute for Theoretical Physics, Russian Academy of Sciences, 142432 Chernogolovka, Russia and [5]National Research University Higher School of Economics, 101000 Moscow, Russia

Email: Ilgiz A. Garifullin* - ilgiz0garifullin@gmail.com

* Corresponding author

Fabrication of the SSV heterostructures

The SSV effect has been studied for a set of samples MgO(001) CoO_x/Py/Cu/HA/Cu/Pb. Here, MgO(001) is a high-quality single-crystalline substrate, the cobalt oxide antiferromagnetic (AFM) layer plays the role of a bias layer, which pins the magnetization of the F1 layer; Py and HA stand for the ferromagnetic F1- and F2-layers; Cu is a normal metallic N-layer, which decouples the magnetizations of F1- and F2-layers; finally Pb is an S-layer. The deposition of layers was performed using a combination of sputtering (for HA) and classical electron-beam evaporation in ultrahigh vacuum (UHV) with a pressure of 10^{-9} mbar (for Co, Py, Cu and Pb). The deposition setup had a load-lock station with vacuum shutters, allowing us to change the sample holder without breaking the UHV in the main deposition chamber. All materials used for evaporation had a purity of better than 4N, i.e., the contamination level could be kept below 0.01 atom %. The thickness of the films during the growth was measured by a standard quartz crystal monitor system. First, the substrates were fixed on a sample holder and transferred into the main deposition chamber through the load-lock station. We used a rotating wheel sample holder in order to prepare a set of samples with different layer sequences in a single vacuum cycle. The deposition of CoO_x was performed in two steps: (I) metallic Co was deposited on the substrate; (II) the substrate was moved into the load-lock station and exposed to 100 mbar of O_2 gas for 2 h. After that, the sample holder was again transferred into the main deposition chamber for the deposition of Py and Cu layers. Subsequently, the sample holder was transferred into the additional chamber for evaporation of the Heusler alloy using sputtering. The HA film was sputtered on the Cu layer at room temperature. Then the sample holder was moved to the main deposition chamber for evaporation of Cu and Pb layers. Finally all samples were covered by a Si_3N_4 protective layer against oxidation. We used the following deposition rates: 0.5 Å/s for Co, Py and Cu films; 0.37 Å/s for HA films; 12 Å/s for Pb films.
